# The knowledge, attitude and behavior on the palliative care among neonatal nurses: what can we do

**DOI:** 10.1186/s12904-024-01470-y

**Published:** 2024-07-03

**Authors:** Yilan Yan, Jiahui Hu, Fei Hu, Longyan Wu

**Affiliations:** Department of Neonatology, Children’s Hospital of Nanjing Medical University, No. 72, Guangzhou Road, Gulou District, Nanjing, Jiangsu Province China

**Keywords:** Knowledge, Attitude, Behavior, Palliative care, Neonate, Nurse

## Abstract

**Background:**

Neonatal nurses should provide timely and high-quality palliative care whenever necessary. It’s necessary to investigate the knowledge, attitude and behavior of palliative care among neonatal nurses, to provide references and evidences for clinical palliative care.

**Methods:**

Neonatal intensive care unit (NICU) nurses in a tertiary hospital of China were selected from December 1 to 16, 2022. The palliative care knowledge, attitude and behavior questionnaire was used to evaluate the current situation of palliative nursing knowledge, attitude and behavior of NICU nurses. Univariate analysis and multivariate logistic regression analysis were used to analyze the influencing factors.

**Results:**

122 nurses were finally included. The average score of knowledge in neonatal nurses was 7.68 ± 2.93, the average score of attitude was 26.24 ± 7.11, the score of behavior was 40.55 ± 8.98, the average total score was 74.03 ± 10.17. Spearman correlation indicated that score of knowledge, attitude and behavior of palliative care in neonatal nurses were correlated with the age(*r* = 0.541), year of work experience(*r* = 0.622) and professional ranks and titles(*r* = 0.576) (all *P* < 0.05). Age (OR = 1.515, 95%CI: 1.204 ~ 1.796), year of work experience (OR = 2.488, 95%CI: 2.003 ~ 2.865) and professional ranks and titles (OR = 2.801, 95%CI: 2.434 ~ 3.155) were the influencing factors of score of knowledge, attitude and behavior of palliative care (all *P* < 0.05).

**Public contribution:**

NICU nurses have a positive attitude towards palliative care, but the practical behavior of palliative care is less and lack of relevant knowledge. Targeted training should be carried out combined with the current situation of knowledge, attitude and practice of NICU nurses to improve the palliative care ability and quality of NICU nurses.

## Introduction

Neonatal intensive care unit (NICU) is a centralized ward for the treatment of neonatal critical diseases, which is established for continuous monitoring and timely and effective rescue treatment and nursing of high-risk newborns. Its purpose is to reduce neonatal mortality and promote neonatal growth and development. NICU provides life support for high-risk newborns, including rescue and treatment of neonatal diseases, neonatal respiratory management and premature infant management [1]. Previous studies [2, 3] have shown that the mortality of neonate with NICU can be as high as 8.16%. The illness and death of newborns can bring great pain and psychological burden to their families. Therefore, it is necessary to carry out corresponding palliative care for neonates and their families.

As an extension of high-quality clinical nursing service, palliative care centered on newborns, is the overall behavior of neonatal nursing service, which must be taken care of by a competent and qualified professional nursing team [4–6]. How to improve the knowledge, attitude and behavior ability of NICU nurses in palliative care is a very important issue that the hospital managers and nurses must pay attention to.

As a hot spot of social concern, palliative care for newborns has attracted more and more attention in recent years. Palliative care in China is in the initial state of development. Kain et al. in Australia [7], Wright team [8] and Kyc team [9] in the United States, Chen et al. in Taiwan [10], Forouzi et al. in southeastern Iran [11] have used the Neonatal Palliative Care attitude scale to investigate the attitude of NICU nurses towards the obstacles of providing palliative care for newborns. These studies reported some obstacles, including insufficient staff, lack of ideal implementation environment, technical requirements, parents’ requirements, nurses’ inability to express their opinions, lack of education, lack of effective counseling, and medical staff’s personal values and attitude in palliative care for newborns [12, 13]. In order to understand the obstacles that affect palliative care for newborns, it’s necessary to investigate the knowledge, attitude and behavior of neonatal nurses. The purpose of this survey was to investigate the current situation of nurses’ knowledge, attitude and behavior of palliative care for newborns, and to evaluate the influence of neonatal nurses’ personal and professional characteristics on their knowledge, attitude and behavior, to provide insights to the clinical palliative care for newborns.

Methods.

This study was a cross sectional survey design. The study has been reviewed and approved by the ethics committee of Children’s Hospital of Nanjing Medical University (No. 202304068-1). And written informed consents had been obtained from all the nurses.

According to the principle of the application of psychometrics, the sample size in the survey is related to the number of variables, and the sample size should be about 10 to 15 times the number of variables, in order to improve the stability of the questionnaire structure and facilitate factor analysis [14]. The number of items used in this survey was 6, and 15 times the number of items waws taken as the sample size of the survey, and then considering the 20% loss rate and invalid questionnaire, the required sample size is 6* 15*1.2 = 108. Therefore, at least 108 nurses should be included in this survey.

In this study, targeted NICU nurses in a tertiary hospital of China were surveyed from December 1 to 16, 2022. The inclusion criteria of nurses in the survey were as follows: formal employees of NICU; whose age was ≥ 18 years old, the nurses knew and agree to participate in this survey. Exclusion criteria: trainee nurses, nurses in other department, volunteers.

We designed a general information questionnaire, including gender, age, nationality, professional title, working years and so on. In addition, we used the knowledge, attitude and measured performance questionnaire [15] to evaluate the current situation of palliative nursing knowledge, attitude and behavior of NICU nurses. The questionnaire consists of 40 items, which is divided into three dimensions: knowledge, attitude and behavior. Among them, there are 20 items in the knowledge dimension, with a score of “1” for correct answers and a score of “0” for wrong or unknown answers. The higher the score, the better the nurses’ knowledge. There were 12 items in the attitude dimension, which were scored by Likert-5 points (1 = strong disagreement, 2 = disagreement, 3 = neutral, 4 = consent, 5 = strong agreement). The higher the score, the better the nurses’ cognitive attitude. There were 8 items in the behavior dimension, which were scored by Likert5 points (1 = almost none, 2 = less, 3 = uncertain, 4 = able to do, 5 = often). The higher the score, the more palliative nursing behaviors of nurses. The Cronbach α coefficients of the questionnaire were 0.758, 0.794 and 0.910, respectively [16]. The internal consistency of the questionnaire was good to evaluate the level of knowledge, attitude and behavior of palliative care of nurses.

We explained the purpose, significance and matters needing attention of this survey to the NICU nurses. After the respondents signing the informed consent form, the investigators distributed the questionnaire uniformly. We collected it on the spot after the nurse filled out the questionnaire.

SPSS 23. 0 software was used for statistical analysis, the counting data were described by frequency and percentage, the measurement data were expressed by mean ± standard deviation, and the t and F tests were used between groups. Univariate and multivariate logistic regression analysis were used to analyze the influencing factors. Spearman correlation analysis was used to analyze the correlation between nurses’ characteristics and their knowledge, attitude and practice. In this study, *P* < 0. 05 as the difference between groups was statistically significant.

## Results

A total of 122 nurses were included. As presented in Table 1, the included nurses were mainly female, most of them were under the age of 35, their academic qualifications are mainly bachelor’s degree, their working years were less than 10 years, and their professional titles were mostly junior and senior nurses.

**Table 1:** The characteristics of included nurses

Characteristic	Cases(*n* = 122)	Percentage
Gender		
Female	111	90.98%
Male	11	9.02%
Age(y)		
18 ~ 30	40	32.79%
31 ~ 35	48	39.34%
> 35	34	27.87%
Education level		
Junior college degree	45	36.89%
Bachelor degree	75	61.48%
Master degree	2	1.63%
Year of work experience		
< 5	39	31.87%
5 ~ 10	36	29.51%
11 ~ 20	37	30.33%
> 20	10	8.19%
Professional ranks and titles		
Junior nurse	31	25.41%
Senior nurse	56	45.90%
Nurse in charge	38	31.15%
Deputy chief nurse	5	4.11%
Chief nurse	2	1.63%
Marital status		
Unmarried	52	42.62%
Married	70	57.38%

As presented in Table 2, The average score of knowledge in neonatal nurses was 7.68 ± 2.93, the average score of attitude was 26.24 ± 7.11, the score of behavior was 40.55 ± 8.98, the average total score was 74.03 ± 10.17.

**Table 2:** The average score of knowledge, attitude and behavior of palliative care for neonatal nurses

Items	Average score	Minimum score	Maximum score
Knowledge section	7.68 ± 2.93	3	14
Attitude section	26.24 ± 7.11	18	39
Behavior section	40.55 ± 8.98	28	51
Total score	74.03 ± 10.17	55	8

As indicated in Table 3, there were significant differences in the score of knowledge, attitude and behavior of palliative care in nurses with different age, year of work experience and professional ranks and titles (all *P* < 0.05). No significant differences in the score of knowledge, attitude and behavior of palliative care in nurses with different gender, education level, and marital status were detected (all *P* > 0.05).

**Table 3:** The score of knowledge, attitude and behavior of palliative care in neonatal nurses with different characteristics

Characteristic	Cases(*n* = 122)	Knowledge				Attitude				Behavior		
		Score	t/F	P		Score	t/F	P		Score	t/F	P
Gender			1.113	0.125			5.204	0.152			6.054	0.137
Female	111	7.67 ± 2.61				26.41 ± 7.75				39.81 ± 8.03		
Male	11	7.72 ± 2.58				26.15 ± 7.81				40.16 ± 9.19		
Age(y)			3.114	0.030			4.125	0.021			5.351	0.014
18 ~ 30	40	6.49 ± 2.58				25.04 ± 7.87				37.36 ± 9.05		
31 ~ 35	48	7.66 ± 2.91				26.14 ± 7.53				40.22 ± 9.85		
> 35	34	8.84 ± 2.65				27.60 ± 7.17				41.43 ± 10.18		
Education level			2.126	0.098			5.102	0.143			6.312	0.057
Junior college degree	45	7.22 ± 2.48				26.13 ± 7.86				39.05 ± 9.64		
Bachelor degree	75	7.69 ± 2.50				26.21 ± 7.92				40.75 ± 9.21		
Master degree	2	7.71 ± 2.36				26.30 ± 8.88				41.83 ± 8.75		
Year of work experience			1.486	0.016			6.078	0.044			7.172	0.036
< 5	39	6.41 ± 2.46				23.13 ± 6.75				38.03 ± 9.54		
5 ~ 10	36	7.59 ± 2.47				24.30 ± 7.94				40.60 ± 8.43		
11 ~ 20	37	7.74 ± 2.55				25.04 ± 6.81				41.01 ± 9.15		
> 20	10	8.83 ± 2.49				25.19 ± 7.05				41.66 ± 9.54		
Professional ranks and titles			3.123	0.041			5.458	0.013			8.146	0.009
Junior nurse	31	6.50 ± 2.85				23.85 ± 8.02				38.04 ± 8.29		
Senior nurse	56	7.62 ± 2.54				24.16 ± 9.13				40.39 ± 9.05		
Nurse in charge	38	7.87 ± 2.61				24.29 ± 8.85				41.92 ± 8.57		
Deputy chief nurse	5	8.07 ± 2.51				25.19 ± 7.72				41.96 ± 9.23		
Chief nurse	2	8.89 ± 2.42				25.35 ± 7.68				42.03 ± 8.84		
Marital status			1.278	0.207			6.794	0.287			8.094	0.114
Unmarried	52	7.66 ± 2.56				23.95 ± 6.81				40.27 ± 9.84		
Married	70	7.68 ± 2.44				24.78 ± 5.99				40.68 ± 8.09		

As presented in Table 4, Spearman correlation indicated that score of knowledge, attitude and behavior of palliative care in neonatal nurses were correlated with the age(*r* = 0.541), year of work experience(*r* = 0.622) and professional ranks and titles(*r* = 0.576) (all *P* < 0.05).

**Table 4:** Analysis of the correlation between total score and characteristics of neonatal nurses

Characteristic	*r*	*P*
Gender	0.143	0.095
Age(y)	0.541	0.021
Education level	0.108	0.167
Year of work experience	0.622	0.015
Professional ranks and titles	0.576	0.037
Marital status	0.113	0.102

As shown in Table 5, multivariate logistic regression analysis indicated that age (OR = 1.515, 95%CI: 1.204 ~ 1.796), year of work experience (OR = 2.488, 95%CI: 2.003 ~ 2.865) and professional ranks and titles (OR = 2.801, 95%CI: 2.434 ~ 3.155) were the influencing factors of score of knowledge, attitude and behavior of palliative care in neonatal nurses (all *P* < 0.05).

**Table 5:** Multivariate logistic regression analysis on the influencing factors of score of knowledge, attitude and practice of palliative care in neonatal nurses

Variables	β	S‾x	OR	95%CI	*P*
Age	0.139	0.101	1.515	1.204 ~ 1.796	0.031
Year of work experience	0.143	0.102	2.488	2.003 ~ 2.865	0.017
Professional ranks and titles	0.149	0.123	2.801	2.434 ~ 3.155	0.014

## Discussions

The results of this study shows that although most nurses recognize the importance of neonatal palliative care, they do not know much about it, which leads to some doubts and deficiencies in their clinical palliative care behavior. There is a lack of standardized palliative care practice guidelines and quality assessment standards and tools, and the contents and items of neonatal palliative care are not standardized [17]. Most nurses have a positive attitude towards the implementation of palliative care for newborns in this study, but it has been reported that some nurse have a negative attitude towards palliative care, mostly worried that after the implementation of palliative care, they will increase their work burden because of insufficient manpower deployment [18, 19]. It is of great significance to find out the promoting factors and obstacle factors that affect nurses’ knowledge, attitude and practice [20]. Most nurses do not have enough knowledge of palliative care, especially for newborns [21]. In addition, palliative nursing education is also inadequate. Most nurses learn very few knowledge and skill of palliative nursing [9, 11].

We have found that neonatal nurses lack of knowledge related to palliative care. The knowledge of palliative nursing is the basis of nursing intervention. The evaluation of palliative care for children by the National Medical institutions Review Committee mainly includes two aspects: one is that medical institutions have the conditions to implement palliative care, and the other is that staff have the ability to implement palliative care to meet the needs of dying children and their families [22, 23]. The hospitals should provide support to nurses to enhance the competence of professionals and apply social support through research and continuing education [24]. The knowledge, skills and hospital conditions of the parents and medical staff are the important factors affecting the implementation of palliative care in NICU, and the nursing staff are the key factors. Therefore, having certain knowledge and ability to implement palliative care is the basis for the implementation of high-quality palliative care, it is necessary to carry out palliative care training in clinical nursing education.

The neonatal nurses have a positive attitude toward the palliative care in this survey. Some studies [25–27] have shown that the implementation of palliative care is related to nurses’ knowledge, attitude, experience and evaluation ability, and these factors will affect the implementation effect. Some studies [28–30] have shown that the attitude of medical staff, whether positive or negative, can have a lasting impact on parents. A positive attitude can help parents alleviate their grief and retain good memories in the process. Not paying attention to finding psychological problems and giving support will make families feel helpless [31]. Therefore, for NICU nurses, they must be able to face the death of neonate with a correct state of mind, and receive systematic palliative education [32, 33]. The continuing education and training of palliative care should attract the attention of the health care providers and managers of relevant universities and medical institutions [34].

The results of this survey show that the palliative nursing behavior of nurses is insufficient. The concept of Chinese traditional culture has brought challenges to the popularization of palliative care in China [35]. The traditional Chinese view of life and death holds that talking about death is a symbol of misfortune and fear, which makes Chinese people afraid to talk about death [36]. This concept is deeply rooted in the hearts of the people, which not only makes family members avoid talking about death, but also brings certain psychological pressure for medical staff to talk about “death” and “palliative care” to parents, which hinders the behavior of palliative care for newborns [37–39]. The cause of palliative care for newborns should be raised to the level of safeguarding human dignity, reducing social burden and reflecting human civilization [40]. In addition, it is necessary to improve the relevant policies and regulations, bring palliative care into the basic medical insurance system, improve the professional quality of nursing care, and strengthen the input of manpower and material resources needed for palliative care [41, 42]. Clinical nursing managers should strengthen the training of palliative nursing knowledge and operation of neonatal nurses and encourage nurses’ palliative nursing behavior.

There are some shortcomings in this survey that deserve careful consideration. First of all, this study is a single-center investigation, the study sample size is small, we cannot perform correlation and multi-factor analysis on specific scores of knowledge, attitude and behavior. Secondly, there are few personal characteristic data of nurses included in this study, and other factors affecting nurses’ knowledge, attitude and behavior of palliative care may not be included in the analysis. The sample size should be expanded in future research. More factors were prospectively included to analyze the current situation and influencing factors of neonatal palliative care, so as to provide evidence support for clinical neonatal palliative care.

## Conclusion

In summary, the results of this survey have indicated that neonatal nurses lack of knowledge related to palliative care and have a positive attitude, but their behavior is relatively insufficient. And age, year of work experience and professional ranks and titles are the influencing factors of score of knowledge, attitude and behavior of palliative care in neonatal nurses. Palliative care is an important part of NICU care. Through the training of nurses’ professional knowledge and skills, NICU nurses’ understanding of palliative care and clinical nursing ability should be effectively improved. Only when nurses correctly understand the connotation of palliative care and the relevant knowledge and skills of palliative care can they provide high-quality palliative nursing care to newborns and their families.

## Data Availability

All data generated or analyzed during this study are included in this published article. The original data will be available from corresponding authors on reasonable request.

## Abbreviations

NICU: neonatal intensive care unit
